# Concurrence of multiple sclerosis and primary biliary cholangitis: Report of 3 cases

**DOI:** 10.22088/cjim.11.2.223

**Published:** 2020

**Authors:** Mozhgan Sattar, Maryam Poursadeghfard

**Affiliations:** 1 Clinical Neurology Research Center, Shiraz University of Medical Sciences, Shiraz, Iran

**Keywords:** Multiple sclerosis, Primary biliary cholangitis, Autoimmune disorders

## Abstract

**Background:**

Multiple sclerosis (MS) is an autoimmune disorder of the central nervous system which damages the myelin and axon. Primary biliary cholangitis (PBC) is a slow progressive liver disease with autoimmune feature in which non-purulent destructive cholangitis and interlobular bile duct destruction occur. Involvement of each of PBC and MS is thought to be related to environmental exposure in genetically susceptible persons.

**Case Presentation:**

Here, we aim to report 3 women 52, 27 and 51 years old with MS and PBC.

**Conclusion:**

Although MS seems to have an association with some autoimmune gastrointestinal disorders such as ulcerative colitis, the concurrence of MS and PBC has been rarely reported.

Multiple sclerosis (MS) is an autoimmune disorder of the central nervous system which damages the myelin and axon. In the early stages of the disease, most cases have a relapsing remitting course with different neurological presentations. This disease could involve multiple domains of the functional systems, such as motor, sensory, vision, cognition, etc.(1). Other autoimmune disorders like rheumatoid arthritis, myasthenia gravis, ulcerative colitis (UC), thrombotic thrombocytopenic purpura and systemic lupus erythematosus (SLE) are more frequent in patients with MS than in the general population (2). Primary biliary cholangitis (PBC) is a slow progressive liver disease with autoimmune feature in which non-purulent destructive cholangitis and interlobular bile duct destruction occur. Like MS, other autoimmune disorders as thyroid diseases, Raynaud and Sjogren syndromes are seen more frequently in PBC. Involvement of each of PBC and MS is thought to be related to environmental exposure in genetically susceptible persons (2, 3). Although MS seems to have an association with some autoimmune gastrointestinal (GI) disorders such as UC (4, 5), the concurrence of MS and PBC has been rarely reported. Here, we aim to report 3 women with MS and PBC. In 2 of them, PBC had been diagnosed years before MS, and in the third one, MS diagnosis had preceded the PBC presentations. As these 2 diseases are both believed to have autoimmune bases, reporting these patients and other similar cases might help to find if there is any association between MS and PBC.

## Cases presentation

**Case 1: **The patient was a 52-year old lady presented with an acute onset of blurred vision in right eye and painful eye movement since a couple of days prior to hospital admission in 2017. She complained of unilateral headache and paresthesia of the left side of the body, as well. In the past medical history, she had diabetes mellitus, hypertension and hypothyroidism.

She also suffered from PBC which was diagnosed about 3 years before according to nausea, vomiting, fatigue and mild discoloration of the sclera. In para-clinic evaluation, a rise in the liver enzymes, alkaline phosphatase and bilirubin levels was detected. Finally, in further evaluation, magnetic resonance cholangiopancreatography (MRCP) showed mild dilatation of the common hepatic and common bile ducts with slightly dilated intrahepatic bile ducts because of intrahepatic bile duct stricture representation of the mild form of PBC. On admission, neurological examination revealed a decrease in the visual activity of the right eye to 50 centimeters for count finger besides the positive relative afferent pupillary defect (RAPD) (Marcus Gunn pupil sign). The muscle power was 4/5 in the left side with upward plantar reflex. In paraclinical evaluation, visual evoked potential (VEP) showed prolonged latency and decreased amplitude of P100 wave in the right eye. Moreover, optical coherent tomography (OCT) of the right eye was outside the normal limit. Perimetry also showed a centrocecal scotoma. 

**Figure 1:**
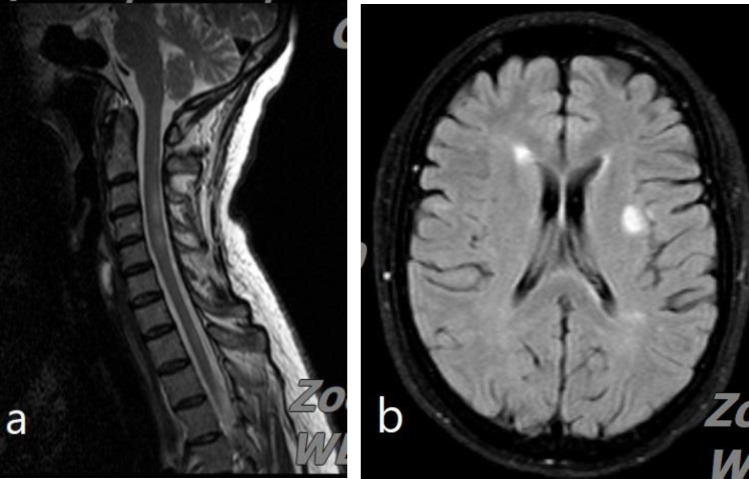
The sagittal view of the cervical and axial view of the brain MRI showed hyperintense lesions. The rest of the imagines are not shown here

Cervical spine MRI showed multiple hypersignal lesions in the level of C3, C4, C5, C7 and T1 with a gadolinium enhancement in the level of C4 and C5 (Figure 1a).

In the brain magnetic resonance imaging (MRI), multiple hypersignal lesions in the periventricular and juxtacortical white matter and the right side of the pons were seen. Furthermore, one gadolinium enhancing lesion measuring 9×9 mm, suggestive for an active lesion, was detected (figure 1b).

Anti-aquaporin 4 antibody was negative. With the impression of MS, she received methyl prednisolone and her visual acuity improved. Subcutaneous glatiramate acetate was started every other day and she was discharged in a good condition. During the next year follow up, she experienced 2 new attacks of MS relapse despite the disease modifying treatment. 

**Case 2: **She was a 27-year-old healthy woman who presented with refractory vertigo, ataxia and blurred vision for the first time 10 days before hospitalization. Her past and drug histories were negative.

In neurology examination, although she was completely oriented to the environment in the first days of the start of the problems, she progressed to the worsening signs and symptoms quickly. Left side weakness with muscle power 3/5, and right side limb dysmetria and tremor were the main neurological abnormality. 

**Figure 2:**
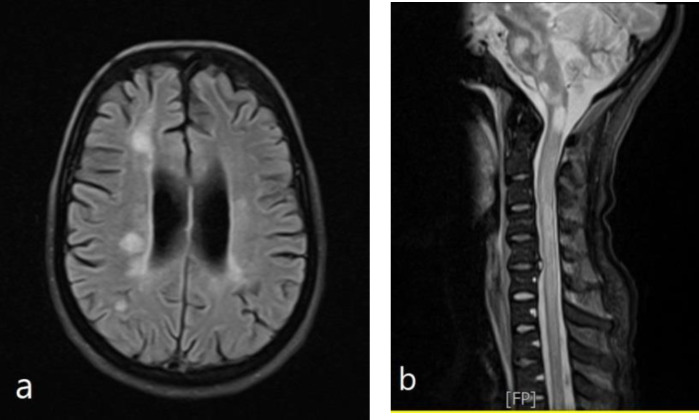
Axial and sagittal views of the brain and cervical MRI of the second case

After admission in the neurology ward, brain MRI was done for her. The imaging had multiple hypersignal tumefactive lesions in the periventricular area with typical feature for MS diagnosis. These lesions involved the subtentorial region including midbrain, pons, and middle cerebellar peduncles. After contrast injection, some of them underwent gadolinium, especially in the supratentorial white matter (figure 2a). Cervical MRI showed a hypersignal lesion extending from C4 to C6 (figure 2b).

NMO-anti body, ESR, CRP, vacuities and tumor markers were all negative. Due to active and widespread plaques above, she received 7 grams intravenous methylprednisolone without acceptable response; then, plasma exchange for 5 sessions was done every other day. Again, there was no improvement; finally, one gram intravenous cyclophosphamide was prescribed to control the patients' situation. After a long time of hospital admission, she was discharged with incomplete recovery of the attack and impression of Marburg acute MS. 2 months later, she developed yellowish discoloration of the skin and sclera besides the rise in the liver enzyme and alkaline phosphatase. Thus, abdominal sonography, CT scan and MRCP were done. MRCP was reported as the "mild prominence of the left intrahepatic biliary tracts without involvement of the right lobe”. This finding could be due to the stricture at the site of the left main hepatic duct. Also, there were multiple small cystic structures, scattered in both lobes, especially in the right lobe which were probably bilomas or hematoma. Due to the border irregularity of the intrahepatic biliary tracts, especially in the right lobe, the possibility of cholangitis should be considered. Finally, endoscopic retrograde cholangiopancreatography (ERCP) and liver biopsy documented PBC. Unfortunately, the patient died in less than one year after the beginning of the disease.

**Figure 3:**
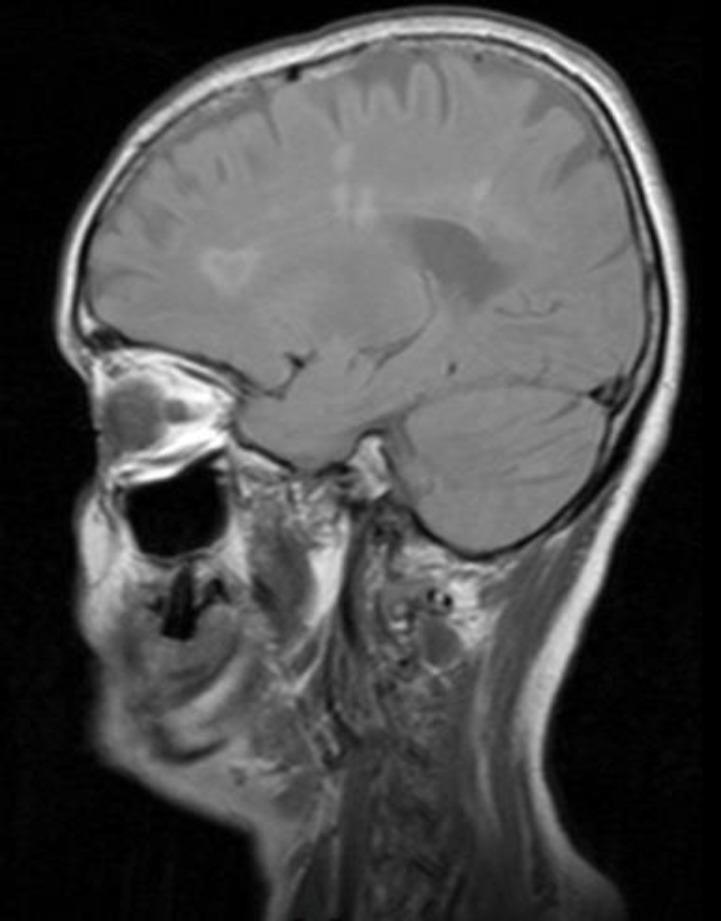
The sagittal FLAIR imaging of the brain MRI showing periventricular hypersignal lesion

**Case 3: **This patient was recognized in out-patient neurology clinic. She was a 51-year-old lady who had developed uveitis attacks about 20 years ago and Behcet disease was diagnosed for her. Since then, she was relatively well till 8 years ago when she developed pruritus and jaundice. In the work up, she had elevated alkaline phosphatase level, and liver needle biopsy showed destructive granulomatous cholangiopathy suggestive of PBC. In 2017, she presented with true vertigo and paresthesia of both upper extremities; therefore, brain MRI was done for her that showed multiple hypersignal lesions in the periventricular and juxtacortical area and also corpus callosum with 2 gadolinium enhancing lesion in favour of MS. The lesions were more typical for MS than Behcet disease, because of abutting the ventricles and gray matter of the cortex. Cervical MRI was normal (figure 3).

## Discussion

Our patients presented with PBC, an autoimmune type of GI problem, which has rarely been reported in the literature. In 1992, the third case of MS and PBC since 1989 was reported. Her symptoms started with optic neuritis and after that she developed PBC. (6). Another male patient with the same diseases was reported in 2003 (7). In these 2 cases, MS preceded PBC; in contrast, 2 of our three cases developed MS after PBC. Based on other reports, MS could precede or follow other autoimmune diseases (2). 

Actually, MS as an autoimmune disease, can coexist with other autoimmune diseases such as SLE, rheumatoid arthritis, chronic active hepatitis, type 1 DM, uveitis, pemphigus, psoriasis, inflammatory bowel disease (IBD) and autoimmune thyroiditis (8). Some reports have revealed that the patients with MS and other autoimmune diseases have less disability and milder form of the disease. (2, 9). Autoimmune diseases are caused by a defect in the human immune system characterized by inability to recognize their own antigens and by a pathological response against these antigens. Both humoral and cell-mediated immunity abnormalities have been reported. Rarely, a few autoimmune diseases coexist in one person which can suggest a similar pathogenetic mechanism. It is estimated that 25 percent of patients with autoimmune diseases have a tendency to develop other autoimmune diseases (10). 

There are a few reports about the association between MS and some GI problems such as IBD, especially ulcerative colitis (UC), but reported cases about the association of MS and PBC are rare. In 2007, Pokorney and co-workers reported 4 patients with MS and UC. In their study and among their patients, they did not find any association between MS and Crohn’s disease (CD) (11). 

In another report in 2004, a patient with UC developed MS 5 years after the remission of her previous disease (12). Moreover, Kimura et al. carried out a retrospective study from 1950 to 1995 on patients who had IBD and definite MS. They could identify 4 people (3 UC and 1 CD) with mild neurologic disease and disability (9). This is in contrast to at least 2 of our patients that had a non-benign course of MS. Sahraian et al. also reported a modified disease course when accompanied by the other autoimmune disorders (2). In 2013, Kowalec et al. identified an unmasked autoimmune hepatitis and primary biliary cirrhosis after interferon-beta in a multiple sclerosis patient (13). Both MS and PBC are chronic inflammatory autoimmune diseases with humoral and T cell activation that destroy the involved tissue. In MS disease, adaptive immune system is activated by CD4 and CD8 T lymphocytes that are harmful to the myelin and nerves within the CNS. Indeed, the role of antibody dependent and antibody-independent B cell was demonstrated (14). PBS is also presented by activation of B and T cell and could frequently accompany other autoimmune diseases (3). 

There are some suggestions indicating that co-existence of autoimmune diseases might be due to the same gene susceptibility and common environmental risk factors (2). In a survey in 2017, a strong genetic relationship between MS and PBC was reported. In this study, fourteen sequence variants associated with MS were studied. In this mete-analysis, the authors discovered that MS- polygenic risk scores (PRS) picked the risk of primary biliary cirrhosis up and vice versa. PBC-PRS causing double risk of PBC increased the risk of MS by 29% and MS-PRS corresponding to the double risk of MS increased the risk of PBC by 81%. They also found out that the severity of MS was actually related to factors other than those determined by genetic susceptibility to the disease onset (15). While MS sometimes occur with other rare autoimmune diseases and it could change the treatment strategy, we think reporting them helps others to identify the occurrence of such disorders earlier and make the best management protocol.
